# Granularity paradox: how emotion taxonomies shape GPT-5’s affective cognition and human-AI alignment

**DOI:** 10.3389/fpsyg.2026.1786724

**Published:** 2026-03-13

**Authors:** Fa Zhang, Jian Chen

**Affiliations:** 1 School of Management, Guangdong University of Science and Technology, Dongguan, China; 2 School of Business, Beijing Institute of Technology, Zhuhai, China

**Keywords:** emotion taxonomy, GPT-5, granularity paradox, human-AI alignment, systematic bias

## Abstract

**Background:**

Large Language Models (LLMs) have demonstrated exceptional capability in textual emotion detection. However, LLM evaluations often treat the emotion taxonomy—the “cognitive ruler” defining the emotional space—as a neutral background variable. The extent to which taxonomic complexity moderates LLM performance remains underexplored.

**Method:**

This study systematically evaluates the impact of emotion taxonomy on GPT-5’s annotation behavior. We constructed a dataset of 2,848 Chinese Weibo posts. Five human annotators and GPT-5 (zero-shot) labeled the data across five distinct taxonomies, each with varying levels of granularity: SemEval (4 classes), Ekman (6 classes), Chinese SevenEmotions (7 classes), Plutchik (8 classes), and GoEmotions (27 classes). A rigorous experimental design, including randomized ordering and washout periods, was implemented to minimize sequence effects. By comparing the results of GPT-5 and manual annotation, the analysis is conducted across three dimensions: performance, consistency, and bias patterns.

**Results:**

Results reveal a significant “granularity paradox”: GPT-5’s performance is strongly negatively correlated with taxonomic complexity, with performance collapsing in fine-grained settings (GoEmotions). Crucially, we identified systematic misalignment mechanisms: (1) Consistency decay: Human-AI agreement significantly deteriorates as semantic boundaries blur in complex taxonomies; (2) Hyper-sensitivity bias: GPT-5 exhibits a tendency to over-interpret neutral texts as emotional, with false-positive rates increasing with taxonomy size; and (3) Arousal shift: The model consistently misclassifies low-arousal negative emotions (e.g., sadness) as high-arousal prototypes (e.g., fear/anger), reflecting a valence-based rather than nuance-based inference logic. Notably, the indigenous SevenEmotions did not yield superior cultural alignment compared to Western taxonomies.

**Conclusion:**

Our findings suggest that emotion taxonomies function as a critical hyperparameter that shapes the cognitive boundaries of GPT. While GPT shows promise, its reliability is compromised by complex taxonomies. Researchers must balance granular detail against model robustness when deploying LLMs for psychological analysis.

## 1 Introduction

Textual emotion detection is a key method for understanding people’s psychological states and social trends. By identifying emotions within massive social media data, researchers can gain valuable insights (Chakraborty et al., 2020). Emotion analysis is now widely used in various fields. For example, it helps in assessing mental health (Kumar et al., 2022), monitoring psychological problems (Aragón et al., 2023), and managing online public opinion (Chu et al., 2024; Field et al., 2022; Li et al., 2024; Zhang et al., 2023).

In recent years, large language models (LLMs) have advanced rapidly (Thapa et al., 2025), demonstrating significant advantages in emotion analysis (Altun and Dörterler, 2025) and becoming mainstream tools for automated annotation. However, emotion annotation is not a singular, objective conversion process; it heavily relies on a predefined emotion taxonomy. Emotion taxonomy is the classification framework that maps affective expressions into discrete categories. Whether emotions can be categorized has long been debated. The discrete approach posits that emotions can be divided into several mutually exclusive categories. Applying the discrete emotion approach for annotation requires selecting an emotion taxonomy. Yet many taxonomies exist, differing significantly in the number of emotion categories, semantic boundaries, and cultural contexts. For human annotators, the complexity of taxonomy directly impacts cognitive load and annotation consistency. The influence of emotion taxonomy on LLMs cannot be overlooked.

Despite LLMs’ outstanding performance in affective computing, systematic research remains scarce on how emotion taxonomy—as cognitive frameworks—influences LLMs’ behavior. For example, does the choice of emotion taxonomy affect LLMs’ emotion analysis performance? Furthermore, does it impact human-AI alignment, or could it induce specific biases? When processing non-English texts, a related question arises: do localized emotion taxonomies hold advantages over mainstream Western taxonomies? Resolving these interconnected questions is crucial for enhancing alignment between LLMs and humans in emotion analysis tasks.

This study evaluates GPT-5 using Chinese Weibo posts, constructed with a benchmark dataset through manual annotation by five annotators. We systematically compare five emotion taxonomies—SemEval, Ekman, Chinese Seven Emotions, Plutchik, and GoEmotions—varying in granularity and cultural characteristics, assessing GPT-5’s behavior across classification performance, consistency, and bias patterns.

The primary contributions of this paper are as follows:

1. Revealing the “Granularity Paradox”: It was discovered that the complexity of emotion taxonomies inversely correlates with GPT-5’s classification performance, demonstrating that expanding the semantic space significantly increases the model’s classification difficulty.
2. Quantifies the boundaries of human-machine consistency: Through multiple consistency metrics and statistical tests, it confirms that complex taxonomies exacerbate cognitive divergence between human and machine annotations, revealing model limitations in handling ambiguous semantics.
3. Identified systemic biases: Discovered two robust classification errors—"Neutral misclassification” and “Sadness shift” and analyzed their underlying logic using psychological archetype theory.

This research not only provides empirical evidence for selecting appropriate emotion taxonomy in emotion analysis but also offers insights for further optimizing LLMs’ performance in complex, cross-cultural psychological tasks.

The remainder of this paper is organized as follows. Section 2 reviews related work. Section 3 describes the dataset and annotation process. Section 4 presents the performance evaluation results, explores the consistency between GPT and the annotators, and reveals the main bias patterns of GPT-5. Section 5 provides discussion, and Section 6 concludes the paper, and Section 7 outlines the limitations of this study and future research directions.

## 2 Related work

### 2.1 Emotion taxonomies as cognitive frameworks

Emotion is a complex psychological phenomenon, and there remains disagreement about emotion (Lerner et al., 2015). Two opposing approaches exist concerning whether emotions can be classified. The dimensional approach posits that emotions are distributed across a continuous spectrum and cannot be simply divided into independent basic emotions. The discrete approach maintains that emotions can be categorized into distinct, finite basic emotions (Brady, 2021; Harmon-Jones et al., 2017). Discrete emotion models provide a clear taxonomy for emotion classification and are widely applied in the field of natural language processing (NLP).

Emotion taxonomy is not merely a collection of labels but a cognitive framework that defines emotional boundaries and organizes psychological experiences. Researchers have proposed various emotion taxonomies. Ekman established six basic emotions—happiness, sadness, anger, fear, disgust, and surprise (Ekman, 1992)—based on physiological foundations and cross-cultural consistency, which gained widespread adoption. Some scholars have refined Ekman’s taxonomy. SemEval-2018 Task 1 streamlined this to four core emotions: anger, fear, joy, and sadness (Mohammad et al., 2018), providing a highly distinguishable engineering standard for NLP tasks. Other taxonomies incorporate additional emotions; for instance, the OCC model expands Ekman’s six basic emotions by adding 16 more, totaling 22 emotion categories (Ortony et al., 1990).

Some scholars posit a hierarchical structure for emotions. Plutchik’s wheel of emotions model features eight (four pairs) fundamental bipolar emotions: joy-sadness, anger-fear, trust-disgust, and surprise-anticipation (Plutchik, 2001). Emotions are categorized into three intensity levels, with two adjacent primary emotions combining to form composite emotions. Parrott proposed a more refined three-tiered model with six primary emotions: love, joy, anger, fear, sadness, and surprise. These primary emotions are further subdivided into secondary and tertiary emotions (Parrott, 2001).

With the rise of social media, traditional emotion taxonomies have shown limitations in capturing nuanced emotions. Demszky et al. (2020) introduced GoEmotions, encompassing 27 emotions that can delicately depict human psychological states during social interactions. GoEmotions stands as the largest and most granular discrete emotion annotation framework. Additionally, datasets like ISEAR incorporate social emotions such as shame and guilt for specific research purposes (Scherer and Wallbott, 1994).

Emotions are not only biologically universal but also culturally constructed. Emotion annotation is also a culturally influenced cognitive activity. As a cornerstone of traditional Chinese psychological thought, the “Seven Emotions Theory” (SevenEmotions) defines seven emotions: 喜 (joy), 怒 (anger), 哀 (sadness), 惧 (fear), 爱 (love), 恶 (disgust), and 欲 (desire) (Sheng, 2013; Wang, 2020). Compared to Western systems, it incorporates emotions like “爱 (love)” and “欲 (desire)” with distinct interpersonal and ethical connotations, aligning more closely with emotional expression in the Chinese context.

In the emotion analysis task, researchers often implicitly adopt a specific taxonomy without systematically comparing how differing taxonomies shape model cognition. As the “ruler” for measuring emotions, does the inherent complexity and classification logic of emotion taxonomy impact model performance? This question remains unanswered.

### 2.2 The capabilities and limitations of LLM emotion analysis

Textual emotion analysis has undergone a paradigm shift from rule-based to data-driven approaches. Early methods primarily relied on lexicon-based techniques, using manually defined rules to classify emotions. With the rise of machine learning and deep learning, machine learning/deep learning-based methods gained prominence. In recent years, LLMs have rapidly evolved, demonstrating outstanding performance across multiple NLP tasks. Leveraging massive parameters and pre-training on large-scale corpora, LLMs demonstrate exceptional semantic understanding and zero-shot learning capabilities, enabling them to capture subtle emotions within text (Kaddour et al., 2023; Zhang W. et al., 2024). GPT has demonstrated the potential to surpass traditional deep learning models in multiple affective computing tasks (Amin et al., 2024).

Researchers have evaluated LLMs’ emotion analysis performance, yielding two contrasting perspectives. Some studies suggest that LLMs’ emotion recognition capabilities are approaching those of human annotators. GPT-4 demonstrates high classification performance across multiple datasets (Liu et al., 2024). Niu et al. (2024) found that GPT-4 achieved recall and macro-F1 scores comparable to human annotations and even outperformed them in certain scenarios. In emotion intensity annotation tasks, GPT’s performance nearly matches models trained on human-annotated data (Bagdon et al., 2024). However, some scholars contend that LLMs lag behind humans when processing complex and nuanced emotional expressions (Koptyra et al., 2023; Qi et al., 2023), and human expertise remains crucial for emotion interpretation (Bojic et al., 2025).

Some studies indicate a “cultural alignment gap” in LLMs’ performance. Despite LLMs’ multilingual capabilities, their performance often significantly declines when processing non-English corpora (Belay et al., 2025). Evaluations on Indonesian tweets (Nasution et al., 2025), German corpora (Greschner and Klinger, 2025), and Persian social media (Tohidi et al., 2025) reveal that, despite strong cross-lingual generalization capabilities, LLM consistency with human annotators remains low when handling metaphors, sarcasm, and culturally specific expressions. This suggests that pre-trained knowledge in LLMs may harbor Western-centric biases, limiting their effectiveness in localized sentiment analysis.

### 2.3 Impact of emotion taxonomy

As the “ruler” of measurement, the attributes of an emotion taxonomy significantly modulate labeling behavior. In psychological research, cognitive load theory indicates that judgment accuracy declines markedly when the number of categories processed exceeds working memory capacity (Sweller, 1988; Baddeley, 2003). This effect has been validated in human emotion annotation tasks: finer granularity and fuzzier semantic boundaries lead to lower inter-annotator agreement (Bostan and Klinger, 2018; Williams et al., 2019). Zhang F. et al. (2024) evaluated different emotion taxonomies, and found that they performed differently in several ways.

However, whether an effect of emotion taxonomy exists for LLMs remains untested. On one hand, LLMs possess vastly superior memory capacity to humans, theoretically enabling them to process large-scale label sets. On the other hand, some studies on machine learning models suggest that fine-grained taxonomy may induce statistical biases toward high-frequency labels (Demszky et al., 2020; Troiano et al., 2023). The choice of emotion taxonomy during dataset annotation impacts the performance and generalization capabilities of machine learning predictors (De León Languré and Zareei, 2024). The semantic density in prompt labels can disrupt LLMs’ zero-shot reasoning (Nasution et al., 2025). Currently, empirical research on how the emotion taxonomy systematically affects LLMs remains scarce. Resolving these questions is crucial for understanding the boundaries of AI’s emotional cognition.

## 3 Materials and methods

### 3.1 Dataset construction and manual annotation

To ensure the ecological validity of the experimental corpus, this study selected Sina Weibo, China’s mainstream social media platform, as the data source. Focusing on hot topics in the socioeconomic domain, a keyword set was constructed for targeted retrieval, initially yielding 3,000 raw posts. Subsequently, rigorous data preprocessing was implemented: commercial advertisements, fragmented texts (<10 characters), and semantically ambiguous noise data were excluded. Ultimately, 2,848 high-quality posts were retained to construct the experimental corpus.

Annotation was performed independently by five systematically trained annotators. To ensure cognitive alignment across different emotion taxonomies, all annotators passed a pre-annotation test. This experiment employed a within-subjects design, where each annotator applied five distinct emotion taxonomies (SemEval, Ekman, SevenEmotions, Plutchik, and GoEmotions) to the same batch of data. All systems included a “Neutral” label to cover samples without distinct emotions.

To control sequence effects and practice effects, we implemented strict randomization and washout strategies:

- Random ordering: The sequence of the five taxonomies for each annotator was randomly generated by the computer.
- Washout period: A minimum 3-day interval was enforced between annotation tasks for adjacent taxonomies.

This design aims to eliminate annotators’ short-term emotional memory of specific corpora, ensuring each annotation is based on independent judgment within the current taxonomy.

### 3.2 GPT-5 annotation

In August 2025, OpenAI released GPT-5, which demonstrated outstanding capabilities across numerous tasks (Singh et al., 2025). To evaluate its emotion understanding capabilities, we developed a Python program that calls the OpenAI API to execute automated annotation tasks. The model identifier is gpt-5-2025-08-07. The experiment employs a zero-shot reasoning paradigm, aiming to simulate baseline model performance in the absence of domain-specific fine-tuning or few-shot examples.

System Role: You are a textual emotion labeling expert.
Task: Annotate Weibo post with emotion by selecting the most appropriate label from the provided label set.
Constrains:
   (1)Label set: {*label_set*}   (2)Must select exactly one label
   (3)Provide a brief justification (<100 characters)
Input: Weibo post: {*text*}Output Format: Emotion:[*Label*];  Justification: [*Reasoning*]

Prompt design adheres to the minimal instruction principle, strictly aligning with human-annotated guidance. We require the model to output only the single best-matching label based on the provided label set, accompanied by a brief justification. The prompt template is as follows:

Regarding parameter configuration, the model version is specified as gpt-5. The *temperature* was set to 0 to ensure reproducibility of results. The *max_tokens* was set to 1,000; it was a deliberate setting based on iterative pilot testing to prevent truncation errors. This buffer allows the model to generate a brief justification (typically 50–100 Chinese characters) alongside the emotion label. We used these justifications to ensure the model’s emotional annotations were based on correct semantic understanding.

Each post was queried once under each emotion taxonomy. Our implementation included a three-time retry logic for each post. A small number of posts failed to return annotation results. In each taxonomy, less than 1% of posts (e.g., due to content filter error) failed to return a valid label (Ke and Watanabe, 2025); these were recorded as ‘null’ and excluded from the analysis.

### 3.3 Ground truth synthesis

Emotions embedded in social media posts often exhibit subjectivity, with different annotators potentially interpreting the emotion of the same text differently. This study proposes a decision strategy to determine the ground truth based on Consensus Level (CL). CL is the maximum number of annotators who assign the same label to a given post. For the five annotators, CL has five levels.

- CL 5 (Unanimous): All five annotators agree on the same label (vote pattern: 5-0-0-0-0).
- CL 4 (Near perfect): Four annotators agree on the same label (vote pattern: 4-1-0-0-0).
- CL 3 (Majority): Three annotators agree on the same label (vote patterns: 3-2-0-0-0 or 3-1-1-0-0).
- CL 2 (Weak majority): The highest vote count for a single label is two. This includes two vote patterns: a single dominant pair (2-1-1-1-0) or a tie between two pairs (2-2-1-0-0).
- CL 1 (Complete dispersion): All five annotators provide different labels (vote pattern: 1-1-1-1-1).

To ensure the reliability of the ground truth, samples categorized as No Consensus (NC) are invalid and excluded from the evaluation. NC includes two scenarios where a unique dominant label cannot be identified: (1) the tied case in CL 2 (vote pattern: 2-2-1-0-0), and (2) the completely dispersed case in CL 1. This filtering mechanism ensures that the evaluation assesses the model’s ability to identify recognizable emotions rather than ambiguous noise. These consensus levels are utilized in Section 4.2.2 to assess the model’s robustness across varying emotional clarity.

### 3.4 Evaluation metrics and statistical tests

We employed a multidimensional metric system to quantify GPT-5’s behavior across three dimensions: classification performance, human-machine consistency, and statistical significance.

(1) Classification performance

Using human ground truth as the benchmark, GPT-5’s annotations are treated as predictions to calculate classification performance metrics. To address the significant class imbalance inherent in social media, we employ two complementary averaging methods—macro-averaging and weighted-averaging—to precision, recall, and F1 scores.

- Accuracy: The proportion of correctly predicted samples relative to the total, reflecting overall classification capability.
- Macro-Precision/Recall/F1: These metrics calculate the precision, recall, or F1 score independently for each class and then take their arithmetic average. By treating each category as equally important regardless of its frequency, macro-averaged metrics effectively highlight the model’s performance on rare categories.
- Weighted-Precision/Recall/F1: These metrics calculate the performance for each category but weigh their contribution based on the percentage of samples. Compared to macro-averaging, weighted metrics provide a more representative measure of the model’s overall performance across the actual ecological distribution of the dataset.

(2) Human-AI consistency

To investigate whether GPT-5 exhibits human-like annotation behavior, two kappa coefficients are computed:

- Group Consistency (Fleiss’ kappa): First, calculate Fleiss’ kappa among 5 human annotators (baseline); then, treat GPT-5 as the sixth annotator and recalculate the metric. A significant decrease in kappa after adding GPT-5 indicates the model disrupts the original human consensus structure.
- Individual Consistency (Cohen’s kappa): Calculate one-on-one Cohen’s kappa between GPT-5 and each of the five annotators to reflect the model’s alignment with individual human annotators.

(3) Significance of differences

The McNemar test is used to assess whether systematic differences exist between GPT-5’s classification error distribution and human annotations. This test focuses on the off-diagonal elements (i.e., inconsistent samples) in the contingency table. If p<0.05, the null hypothesis is rejected, indicating a statistically significant difference in classification decisions between the model and human annotators.

## 4 Results

### 4.1 Analysis of human annotation results

We first analyzed the human annotation results for the 2,848 Weibo posts across the five emotion taxonomies. Each post was independently labeled by five annotators. The labeling options included (1) emotion categories defined within the taxonomy, (2) neutral, and (3) no suitable emotions. Following the ground truth synthesis method, samples that failed to reach consensus were categorized as “NC” (No Consensus). In constructing the evaluation datasets, both “NC” samples and “no suitable emotions” were excluded. The distribution of annotation results across the five taxonomies is presented in Table 1.

**Table 1:** Statistics of human ground truth across five emotion taxonomies.

Emotion taxonomy	NC	No suitable emotion	Valid samples	Majority baseline (neutral)
SemEval	350	217	2,281	58.7%
Ekman	377	140	2,331	58.0%
SevenEmotions	336	131	2,381	59.8%
Plutchik	330	66	2,452	59.1%
GoEmotions	311	1	2,536	49.4%

The data filtration results reveal that the volume of valid samples increased progressively with taxonomic granularity, rising from 2,281 in SemEval to 2,536 in GoEmotions. This trend is primarily driven by the coverage of emotion taxonomies. In the coarse-grained SemEval, 217 samples were categorized as “no suitable emotions” due to a lack of matching emotion. In contrast, this number dropped to 1 in the fine-grained GoEmotions. This indicates that finer-granularity taxonomies enhance the coverage of affective states in social media posts. The number of NC samples remained relatively stable across taxonomies (ranging from 311 to 377), suggesting that human annotators can maintain a consistent level even when faced with an expanded set of options.

For each taxonomy, we calculated the sample size and proportion of each emotional and neutral label (detailed in Appendix Tables S1–S5). Across all taxonomies, the data exhibits a pronounced class imbalance, which is characteristic of naturalistic social media posts:

- Dominance of neutrality: The “neutral” category consistently represents the majority class, ranging from 49.4% (GoEmotions) to 59.8% (SevenEmotions). This distribution establishes a critical Majority Class Baseline (MCB) for evaluating model performance.
- Long-tail distribution: Conversely, many specific emotion categories reside in the “long tail.” For example, in SevenEmotions, indigenous categories such as love” (0.7%), “disgust” (1.0%), and “desire” (0.5%) constitute only a tiny fraction of the dataset. In GoEmotions, this fragmentation is even more extreme, with 13 categories representing less than 1% of the total samples.

### 4.2 Impact of taxonomy on model performance

#### 4.2.1 Overall classification performance

Under the five emotion taxonomies, using human ground truth as the actual values and GPT-5 annotations as the predicted values, we calculated the classification performance metrics: accuracy, precision, recall, and F1 score. The results are shown in Table 2.

**Table 2:** Performance of GPT-5 across five emotion taxonomies.

Emotion taxonomy	Number of emotion categories	Accuracy	Precision		Recall		F1 score	
			Macro	Weighted	Macro	Weighted	Macro	Weighted
SemEval	4	0.6569	0.4792	0.7728	0.6178	0.7174	0.4568	0.7172
Ekman	6	0.6257	0.4619	0.7864	0.5280	0.6628	0.3749	0.6941
SevenEmotons	7	0.6026	0.4098	0.7923	0.5293	0.6348	0.3432	0.6688
Plutchik	8	0.5520	0.3041	0.7960	0.4690	0.5665	0.3141	0.6258
GoEmotions	27	0.4216	0.2325	0.6849	0.3149	0.4156	0.2134	0.4680

Table 2 indicates that the complexity of the taxonomy is a key moderating variable determining model performance. As the emotion granularity expands from SemEval (4 categories) to GoEmotions (27 categories), accuracy drops from 0.6569 to 0.4216, the macro F1 falls from 0.4568 to 0.2134, and the weighted F1 falls from 0.7172 to 0.4680, indicating significant performance degradation.

The number of emotion categories, serving as a measure of taxonomy complexity, exhibits a strong negative correlation with model performance. The Pearson correlation coefficient between the number of emotion categories and accuracy is −0.9651 (p<0.05), while the correlation with macro F1 score is −0.9605 (p<0.05), with the weighted F1 score is −0.9797 (p<0.05).

To provide a robust inferential basis for the relationship between taxonomic granularity and model performance, we employed Generalized Estimating Equations (GEE) regression. This approach was chosen to move beyond simple descriptive trends and to address the statistical requirements of independence and sample-level variance. We transformed the samples in the five taxonomies into a long table consisting of 11,981 observations. Each observation was defined by three variables: (1) Post_id; (2) Taxonomic granularity (4, 6, 7, 8, or 27); and (3) Is_Correct, a binary dependent variable (1 = Correct, 0 = Incorrect). We then applied GEE with a binomial distribution and a logit link function. By grouping observations by Post_id, the GEE model explicitly accounts for the intra-subject correlations inherent in repeated-measures design, thereby providing robust standard errors and unbiased estimates of the effect of granularity on model performance. The result is shown in Table 3.

**Table 3:** GEE regression results.

Variable	Coefficient	Std err	*z*	*p*	Lower	Upper
Intercept	0.8852	0.042	21.136	0.000	0.803	0.967
Granularity	−0.0474	0.002	−24.335	0.000	−0.051	−0.044

The GEE analysis reveals a significant negative effect of taxonomic granularity on accuracy (β=−0.0474,p<0.001). This confirms that the “Granularity Paradox” is not merely a descriptive observation but a statistically robust phenomenon: for every additional category introduced into the taxonomy, the log-odds of the model producing a correct annotation significantly decrease.

Comparing SevenEmotions (7 categories, localized) with Ekman (6 categories, Western universal), their category counts are similar. The accuracy was 0.60620 in SevenEmotion and 0.6257 in Ekman, indicating that SevenEmotion did not outperform Ekman. In SevenEmotion, despite the inclusion of labels like “love” and “desire” that align with Chinese linguistic contexts, the model failed to demonstrate the anticipated cultural advantage. This suggests that GPT-5’s emotional reasoning logic remains primarily driven by Western-dominant schemas embedded in its pre-training, failing to effectively activate the deep semantics of indigenous cultural concepts.

#### 4.2.2 Robustness analysis across consensus levels

Social media posts may have varying degrees of emotional clarity. Emotional clarity represents the extent to which a post conveys a singular, recognizable emotion that can be consistently decoded by human observers. To isolate the impact of emotional clarity on model performance, samples were divided into four groups based on consensus levels (5, 4, 3, and 2). The accuracy across different consensus levels for the five taxonomies is shown in Table 4.

**Table 4:** Accuracy of GPT-5 across five emotion taxonomies at different consensus levels.

Emotion taxonomy	Consensus level			
	5	4	3	2
SemEval	0.8836	0.6621	0.4685	0.3796
Ekman	0.8651	0.7190	0.4739	0.2957
SevenEmotions	0.8550	0.5922	0.4737	0.3177
Plutchik	0.7960	0.5372	0.4000	0.2749
GoEmotions	0.6884	0.3188	0.3258	0.2016

**Figure 1:**
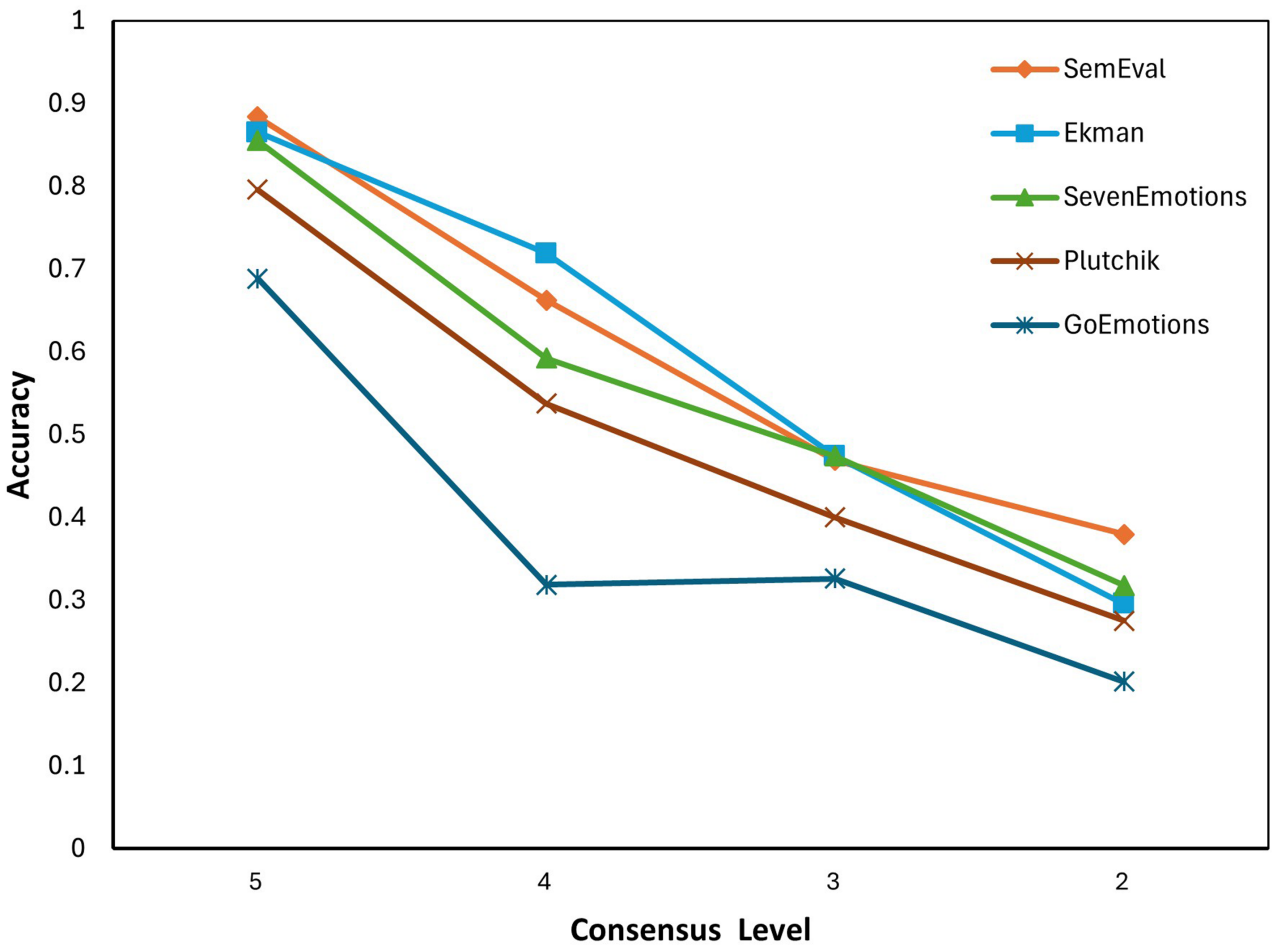
Relationship between accuracy and consensus levels across five emotion taxonomies.

The relationship between consensus levels and accuracy is depicted in Figure 1. The accuracy is significantly positively correlated with the consensus level across the coarse-grained taxonomies (SemEval, Ekman, SevenEmotions, and Plutchik), with Pearson coefficientsr>0.98 (p<0.05). However, for the fine-grained GoEmotions, the correlation did not reach statistical significance (r=0.89,p=0.11). This lack of significance is primarily attributed to a non-linear performance collapse; the accuracy in GoEmotions drops abruptly by 53.7% when shifting from CL 5 to CL 4 and then plateaus at a low baseline (approx. 20–32%) for all lower consensus levels. This suggests that for fine-grained taxonomies, even a minor reduction in emotional clarity (from CL 5 to CL 4) is sufficient to exhaust the model’s discriminative capacity. The performance varies at different CL levels as follows:

- Performance Ceiling (CL 5): For samples with unanimous agreement, coarse-grained taxonomies (SemEval, Ekman, SevenEmotions) achieve accuracy exceeding 85%; the average accuracy is 86.79%, demonstrating that GPT-5 possesses near-human discrimination capabilities for unambiguous emotions. In contrast, the fine-grained GoEmotions lagged significantly, reaching only 68.84%, highlighting the inherent difficulty of fine-grained classification.
- Sensitivity to Clarity (CL 5 to 4): A critical divergence occurs when the consensus level drops from 5 to 4. GoEmotions exhibited a 53.7% relative decrease in accuracy (68.84% → 31.88%), which is significantly steeper than the 25.1% decline observed for SemEval (88.36% → 66.21%).
- The “Floor” Effect (CL 2): For low-clarity samples, SemEval maintained the highest accuracy at 37.96%. Conversely, accuracy for Ekman, SevenEmotions, and Plutchik clustered between 27.49 and 31.77%. GoEmotions recorded the lowest accuracy at 20.16%, representing a 46.9% relative deficit compared to SemEval.

Moving beyond descriptive observations, we employed a mixed-effects logistic regression on the instance-level dataset to quantify the decline rates and test for statistical significance. The model specified Is_Correct as the outcome, with Taxonomic Granularity, Consensus Level, and their interaction as fixed effects, and Post_id as a random effect to account for repeated-measures variance. The result of regression analysis is shown in Table 5.

**Table 5:** Mixed-effects logistic regression results.

Variable	Coefficient	Std err	*z*	*p*	Lower	Upper
Intercept	−1.9434	0.155	−12.542	0.000	−2.247	−1.640
Granularity	−0.0402	0.010	−4.090	0.000	−0.059	−0.021
Consensus level	0.7587	0.040	18.764	0.000	0.679	0.838
Granularity: consensus level	−0.0025	0.002	−1.034	0.301	−0.007	0.002

The regression analysis confirms that consensus level is a robust positive predictor of model performance (β=0.7587,p<0.001). This indicates that regardless of the taxonomy used, GPT-5 is highly sensitive to the emotional clarity; higher human consensus significantly increases the log-odds of a correct prediction.

Controlling for emotional clarity, taxonomic granularity exerts a significant negative impact on accuracy (β=−0.0402,p<0.001). However, the performance degradation follows a non-linear threshold pattern rather than a uniform linear decline.

Crucially, the mixed-effects model tested whether taxonomic granularity is disproportionately more sensitive to clarity. The interaction term between granularity and consensus level was not statistically significant (β=−0.0025,p=0.301).

This result suggests a phenomenon of “parallel decay.” While GoEmotions exhibits the lowest absolute accuracy, the rate at which performance decays as CL decreases is statistically comparable to that of simpler taxonomies. This implies that the “Granularity Paradox” acts as a systemic penalty: it depresses the probability of correct classification uniformly across all levels of CL.

In summary, taxonomic granularity impacts both the upper limit and the lower bound of GPT-5. While the model exhibits a threshold-like drop in performance when scaling to 27 categories, the statistical evidence confirms that the detrimental effect of granularity is omnipresent and independent of the post’s inherent clarity (non-significant interaction).

#### 4.2.3 Micro-analysis of emotion categories

Following the macro-level performance analysis, to deeply explore the internal mechanisms of LLM decision-making, we further analyzed the performance of various emotions at the micro-level. Since the sets of emotion labels across the five taxonomies are not entirely the same, we categorized the emotion labels into three distinct types for separate analysis.

A Core emotion recognition capability

Joy, anger, sadness, and fear are present across the five taxonomies, representing universally applicable core emotions. The F1 score, the harmonic mean of precision and recall, comprehensively reflects a model’s ability to recognize specific emotions. Under the five taxonomies, the macro F1 scores for the four core emotions are shown in Table 6.

**Table 6:** Macro F1 score for four core emotions across five emotion taxonomies.

Emotion taxonomy	Joy	Anger	Sadness	Fear
SemEval	0.619355	0.510949	0.707224	0.462687
Ekman	0.593186	0.481283	0.649446	0.487671
SevenEmotions	0.509158	0.465241	0.682635	0.488323
Plutchik	0.576687	0.453782	0.610390	0.492147
GoEmotions	0.346939	0.566667	0.128571	0.258772

**Figure 2:**
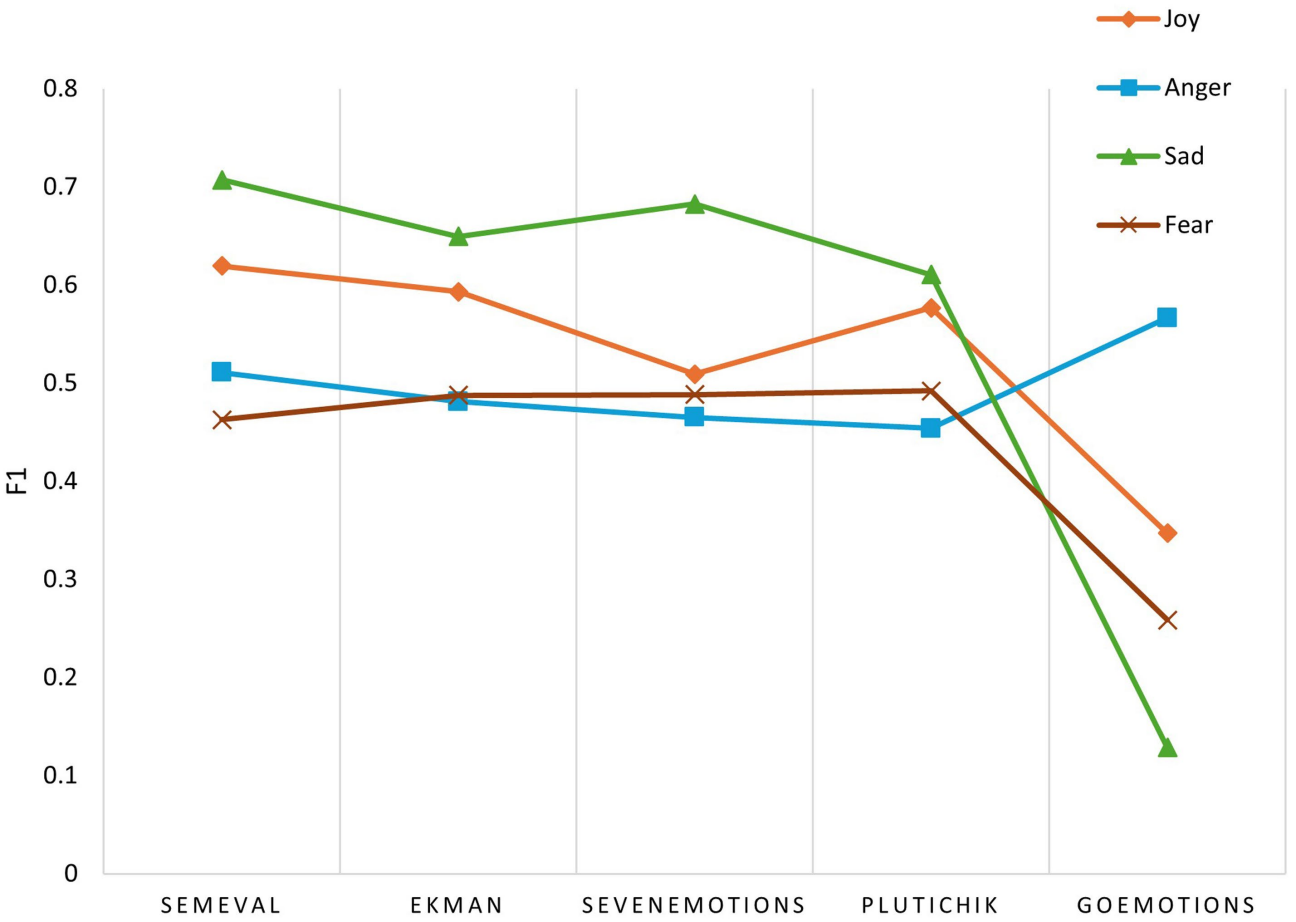
F1 score of the four core emotions under five emotion taxonomies.

As taxonomic granularity increases, the F1 score evolution trajectory for the four core emotions is shown in Figure 2.

Overall, as taxonomy becomes more complex, GPT’s ability to recognize the four core emotions declines. However, the specific manifestations of each emotion vary, revealing two different dynamics.

(1) Semantic dilution effect

For sadness, GPT-5 maintains the highest recognition level under simpler taxonomies. In SemEval, it achieves an F1 score of 0.707 while also sustaining high performance in Ekman, SevenEmotions, and Plutchik (0.610 ~ 0.683). This indicates that sadness, as a core emotion, possesses high linguistic salience, enabling the model to accurately capture sadness in post. However, a precipitous collapse occurs in GoEmotions. The F1 score drops to 0.129, representing an 81.8% relative decrease compared to the SemEval baseline. This primarily stems from GoEmotions subdividing sadness into multiple similar emotions like sadness, grief, and remorse. Under zero-shot learning, GPT-5 struggles to delineate the subtle boundaries between these synonyms, leading to excessive dilution of core semantics and triggering classification failure.

Joy exhibits a pattern like sadness. In simpler taxonomies, its F1 score generally maintains a high level, ranging from 0.5091 to 0.6193. However, in GoEmotions, the F1 score falls to 0.347, a 44.0% relative decline from its peak in SemEval. This decline stems from emotions like amusement and excitement, which are similar to joy, causing the core semantic meaning to become overly diluted and triggering classification failure.

Fear demonstrated high stability across the four simpler taxonomies, with F1 scores fluctuating within a narrow margin (0.463 to 0.492). However, in GoEmotions, the F1 score plummets by 47.4% relative to Plutchik, reaching a low of 0.259. When the number of emotions increases, the model struggles to distinguish between fear and similar emotions like nervousness. This excessive dilution of core semantic meaning triggers classification failure.

(2) Semantic purification effect

Anger exhibits counterintuitive trends. From SemEval to Plutchik, the F1 score shows a gradual decrease of 5.7 percentage points (0.511 → 0.454). However, in GoEmotions, the F1 score unexpectedly rises to 0.567, representing a 24.9% relative improvement over Plutchik and even surpassing the SemEval by 5.6 percentage points. This anomalous phenomenon reveals the semantic purification effect. In coarse-grained taxonomy, anger serves as a broad container encompassing both “annoyance” and “disapproval.” Within GoEmotions, however, the separation of annoyance and disapproval into distinct categories purifies the remaining anger label, refining it into a dedicated term for high-intensity rage. GPT-5 exhibits higher classification confidence toward such sharply defined, narrowly characterized extreme emotions. This finding suggests that for distinctively extreme emotions, fine-grained taxonomies aid models in pinpointing prototypical instances.

B Identification of neutral

In emotion analysis, “neutral” indicates the absence of discernible emotion. The recognition level of neutral serves as a benchmark for measuring whether LLMs produce emotional hallucinations or overinterpret non-emotional text. The macro precision, recall, and F1 score for the neutral across the five taxonomies are shown in Table 7.

**Table 7:** Performance of GPT-5 for “neutral” across five emotion taxonomies.

Emotion taxonomy	Precision	Recall	F1 score
SemEval	0.8978	0.6917	0.7814
Ekman	0.9305	0.6731	0.7811
SevenEmotions	0.9582	0.5992	0.7373
Plutchik	0.9623	0.5399	0.6917
GoEmotions	0.9569	0.5410	0.6912

**Figure 3:**
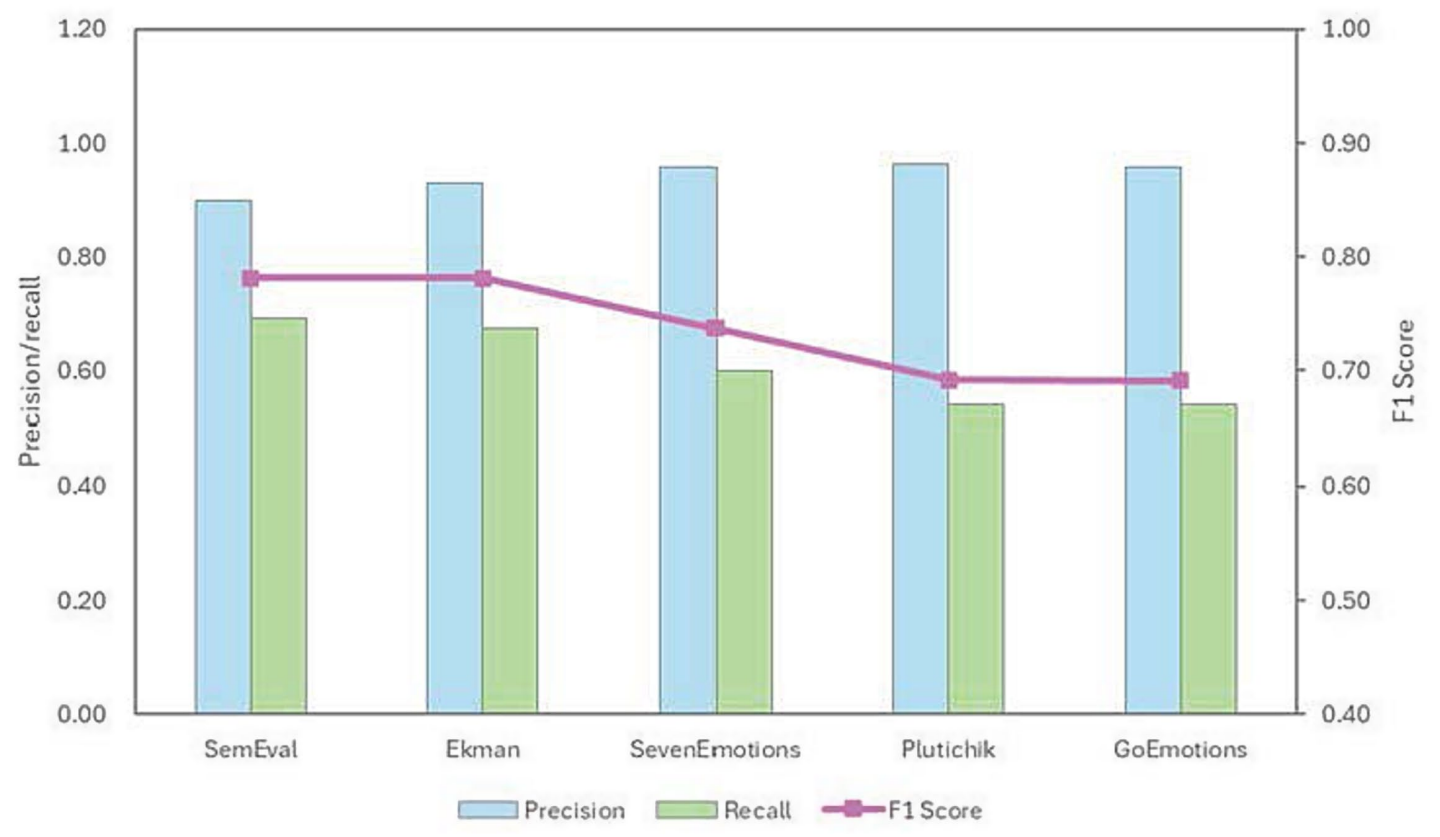
Precision, recall, and F1 score of “neutral” across five emotion taxonomies.

Figure 3 depicts the trends in macro precision, recall, and F1 score as emotion granularity increases.

Across the five taxonomies, the precision for the neutral remained high, ranging from 0.8978 to 0.9623. This indicates that, regardless of the taxonomies employed, GPT-5 achieves exceptional accuracy when labeling posts as neutral. This reflects the model’s significant conservatism in identifying text lacking distinct emotion—it avoids mislabeling clearly emotional statements as neutral. Precision increases with greater granularity, demonstrating that the model applies stricter criteria for defining emotionless text within complex taxonomies.

Recall for neutral is notably lower than precision and decreases with increasing granularity. In SemEval, recall stands at 0.6917, but it decreases by 15.07 percentage points to 0.5410 in GoEmotions. This 21.8% relative decline in recall confirms the presence of “emotional hallucination.” When confronted with an extensive set of labels, the model tends to overinterpret subtle signals in neutral text, forcing samples that should be neutral into specific emotion categories. The more complex the taxonomies, the stronger the incentive for this overinterpretation.

Regarding the F1 score, GPT-5 demonstrates a high level of performance on neutral labels. In SemEval and Ekman, the F1 remains stable at approximately 0.78. However, starting from SevenEmotions, the F1 drops to 0.7373 and ultimately stabilizes near 0.691 (a total decline of 11.5% from the SemEval baseline) in Plutchik and GoEmotions.

Analysis of “neutral” further demonstrates that emotion taxonomy modulates GPT-5’s annotation decisions. Simple taxonomy helps maintain the model’s ability to capture neutral text, while complex taxonomy induces a tendency to assign emotion to neutral text more frequently. These findings alert researchers that while pursuing higher emotion recognition levels, they must be mindful that complex labeling systems can impact a model’s ability to identify neutral text.

C Recognition of social emotions

Emotion taxonomies (excluding SemEval) often include complex emotion categories besides core emotions, such as trust, appreciation, and admiration. Expressions of these emotions frequently lack direct lexical representation and heavily rely on inferences about contextual intent, social interaction, and future states. GPT-5 performs poorly on these emotions, reflecting higher-order cognition and sociality. In Ekman, disgust shows the worst performance with an F1 score of 0.0546. In SevenEmotions, love performs worst with an F1 score of 0.1481. In Plutchik, trust performed worst with an F1 score near zero. In GoEmotions, emotions like admiration and disapproval performed worst, with F1 scores near zero.

This stems from core emotions having strong statistical co-occurrence with specific lexical cues in the pre-training corpus, enabling the model to recognize them well. Emotions like trust and admiration, however, are often implicit within the text’s pragmatic logic. For example, the sentence “I will always support your decision” conveys trust yet contains no explicit emotional vocabulary. This low performance on such emotions reveals a deep limitation in LLMs’ emotional alignment: models currently rely primarily on surface-level semantic matching, lacking the capacity for deep reasoning about social norms, interpersonal intent, and underlying psychological states.

### 4.3 Human-AI alignment

LLMs differ from human emotional understanding mechanisms (Huang et al., 2024). Whether employing different emotion taxonomies impacts the alignment between LLMs and human annotators is a critical issue in LLM evaluation. We assess GPT-5’s alignment with human emotional cognition across three dimensions: group consistency, individual consistency, and statistical significance.

#### 4.3.1 Group consistency

Fleiss’ kappa is a classic metric for evaluating multi-annotator consistency, used to measure relative agreement among multiple annotators. Under the five taxonomies, Fleiss’ kappa was first calculated for five human annotators. Then, GPT-5 was added as the sixth annotator to the group, and Fleiss’ kappa was recalculated. Comparing the two yields the change in Fleiss’ kappa after incorporating GPT. Fleiss’ kappa across the five taxonomies is presented in Table 8.

**Table 8:** Fleiss’ kappa across five emotion taxonomies.

Emotion taxonomy	Fleiss’ kappa (annotators)	Fleiss’ kappa (annotators + GPT-5)	Δ kappa
SemEval	0.4622	0.4576	−0.0046
Ekman	0.3770	0.3826	+0.0057
SevenEmotions	0.3912	0.3893	−0.0018
Plutchik	0.4063	0.3899	−0.0164
GoEmotions	0.4381	0.3755	−0.0625

Based on Fleiss’ kappa for human annotators, the consistency level across the five taxonomies ranges from 0.3770 to 0.4622, falling within the “fair” to “moderate” range (Landis and Koch, 1977). After incorporating GPT, Fleiss’ kappa decreased across four taxonomies (Δkappa < 0), indicating that GPT-5’s emotion judgment logic diverges from human annotators. GoEmotions exhibited the most substantial divergence, with Fleiss’ kappa dropping from 0.4381 to 0.3755—a relative decline of 14.3%, suggesting greater divergence between the model’s emotion judgments and human annotators in fine-grained taxonomy.

Notably, under the Ekman taxonomy, incorporating GPT-5 resulted in a consistency increase of 0.0057 (from 0.3770 to 0.3826). This may suggest that Ekman’s six basic emotions possess high semantic purity within the pre-training corpus, with their classification criteria aligning precisely with the center of human consensus. This implies that the Ekman may represent the lowest-cost approach for human-machine alignment in collaborative emotion annotation tasks.

#### 4.3.2 Individual consistency

To evaluate the validity of the human ground truth and the model predictions, we conducted a dual-layer reliability analysis: assessing both within-annotator self-consistency and inter-annotator agreement. We got the within-annotator self-consistency by measuring how often they assign identical labels to the same post across different taxonomies. Results are detailed in Appendix Table S6. To assess inter-annotator agreement, we calculated pairwise Cohen’s kappa among the five annotators across all five taxonomies. We then computed the average kappa relative to the other four peers to quantify their alignment with the collective consensus; these results are presented in Appendix Table S7.

These results reveal significant heterogeneity in reliability among the human annotators.

- Annotators 2 and 5 exhibited exceptional reliability, with self-consistency rates exceeding 93% and maintaining the highest average pairwise kappa across all taxonomies (e.g., > 0.37 in Ekman).
- Annotator 4 showed the lowest self-consistency (59.61%) and lower agreement with other human peers (e.g., a mean kappa of only 0.238 in the Ekman taxonomy). This multi-dimensional evidence suggests that Annotator 4’s judgments are characterized by high internal entropy and a divergence from the collective consensus.

Cohen’s kappa was further employed to assess the agreement between GPT-5 and human annotators. Under the five taxonomies, Cohen’s kappa between GPT-5 and each annotator is presented in Table 9.

**Table 9:** Cohen’s kappa between GPT-5 and each annotator across five emotion taxonomies.

Emotion taxonomy	Cohen’s kappa					Average
	A1	A2	A3	A4	A5	
SemEval	0.4782	0.4916	0.5018	0.4165	0.3687	0.4513
Ekman	0.4726	0.4528	0.4496	0.2172	0.3851	0.3955
SevenEmotions	0.4193	0.4299	0.4680	0.2811	0.3395	0.3876
Plutchik	0.4001	0.3893	0.4407	0.2362	0.3508	0.3634
GoEmotions	0.3019	0.2992	0.3500	0.1516	0.2455	0.2696

This table indicates that GPT-5’s alignment with human annotation is moderated by the reliability of the annotator and the taxonomic granularity.

- GPT-5 achieved its peak alignment with high-stability annotators. For instance, the average Cohen’s kappa with Annotator 3 (0.4420) and Annotator 2 (0.4124) across all taxonomies significantly outperformed the alignment with others. In the simplest taxonomy, SemEval, the kappa with Annotator 3 reached a maximum of 0.5018, suggesting the model’s decision logic effectively simulates the judgment patterns of stable human annotators.
- Conversely, the model exhibited consistently lower alignment with Annotator 4, with an average kappa of only 0.2605. Notably, in Ekman, the kappa for Annotator 4 dropped to 0.2172, while the other four annotators maintained a mean of 0.4400. Since Annotator 4’s labels deviate significantly from the human consensus, these lower values reflect annotator-specific noise rather than a deficit in the model’s emotional understanding.
- Across all five annotators, alignment levels exhibit a negative trajectory as emotion granularity increases. The average kappa declines from 0.4513 in SemEval to 0.2696 in GoEmotions, representing a 40.3% relative reduction. This confirms that fine-grained taxonomies inherently lower the ceiling for alignment by increasing the complexity of the decision space.

#### 4.3.3 Systemic deviation

To determine whether statistically significant differences exist between GPT-5 and human annotations, a McNemar test was conducted comparing GPT annotations across the five taxonomies with human ground truth. This test aims to assess marginal homogeneity in classification decisions—specifically, whether differences stem from random variation or systematic deviation. Test results are presented in Table 10.

**Table 10:** GPT-5 vs. human annotation McNemar test.

Emotion taxonomy	$\chi^{2}$	*p*-value	Sig.
SemEval	854.0012	<0.001	Yes
Ekman	922.4646	<0.001	Yes
SevenEmotions	995.0010	<0.001	Yes
Plutchik	1140.0009	<0.001	Yes
GoEmotions	1494.0007	<0.001	Yes

Results indicate that across all taxonomies, McNemar’s test yielded *p*-values consistently below 0.001. GPT-5 exhibits a significant, non-random systematic shift relative to human annotations. This signifies a substantial misalignment between the model’s decision logic and human annotators.

The magnitude of this human-machine discrepancy, as quantified by the $\chi^{2}$statistic, demonstrates an intensification as emotion granularity evolves from coarse to fine-grained. The $\chi^{2}$ values increased from 854.00 in SemEval to 1494.00 in GoEmotions. This indicates that the finer the emotion, the more pronounced the divergence between models and humans. The human-machine discrepancy reaches its maximum under GoEmotions, validating that fine-grained taxonomy leads to greater deviation between models and humans.

### 4.4 Classification bias of GPT-5

**Figure 4:**
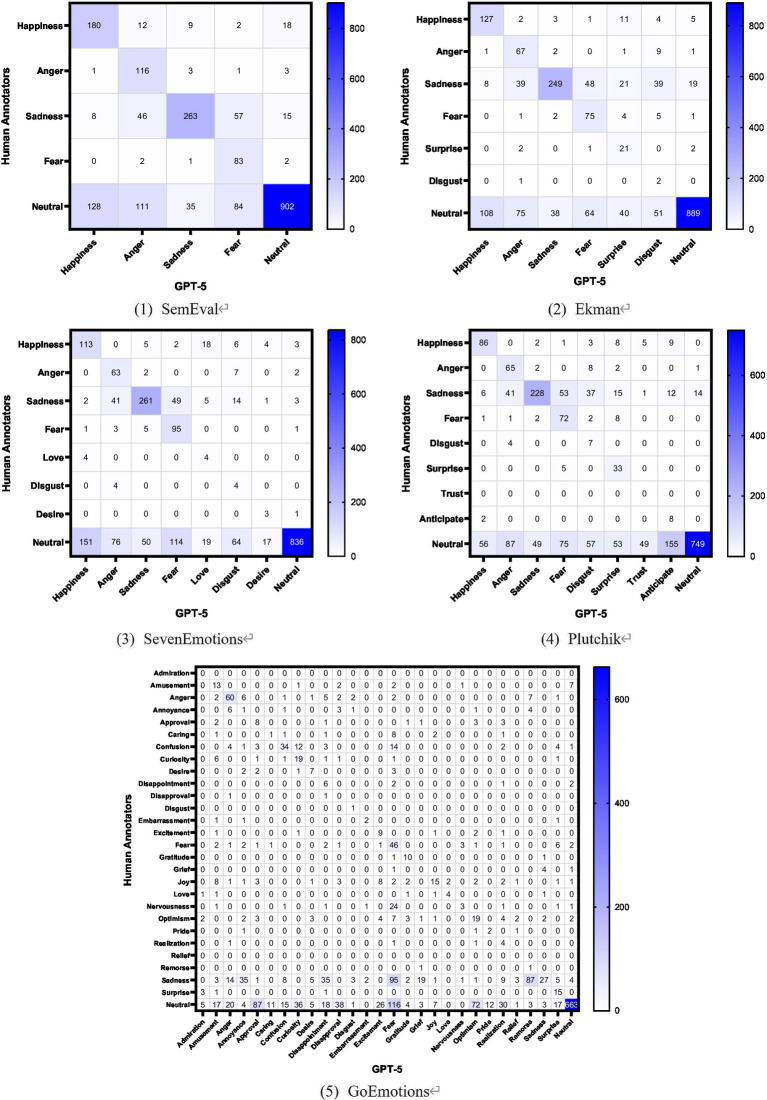
Heatmap of the five confusion matrices.

Given the significant discrepancies between GPT-5 and human annotations, what are the main classification biases? Do these biases vary across different taxonomies? We chose samples with a consensus level ≥3 for analysis. These samples can be assigned a ground truth label through majority voting and serve as reliable true values. By using the human-annotated ground truth as rows and the GPT-5 annotation results as columns, we constructed a confusion matrix. The confusion matrices for the five emotion taxonomies are shown in Figure 4.

The confusion matrices across the five emotion taxonomies reveal two common classification biases, with misclassified samples predominantly concentrated in the “neutral” and “sadness” ground truth labels.

(1) Hypersensitivity to neutrality

GPT-5 labels numerous samples deemed “neutral” by humans with some emotion, representing the most obvious bias with a high misclassification rate. For posts considered objective statements by humans, the model captures extremely subtle lexical cues and amplifies them into explicit emotions. This bias may cause the model to generate emotional “false positives” in practical applications.

As the number of emotional labels increases, GPT-5’s misclassification rate of neutral samples as emotional rises significantly. In SemEval, the misclassification rate is 28.4% (358/1260). In Ekman, it reaches 29.7% (376/1265). In SevenEmotions, the misclassification rate rose to 37.0% (491/1327). In Plutchik, it climbed to 43.7% (581/1330). In GoEmotions, the misclassification rate reached 45.4% (551/1214).

As emotional granularity increases, the model gains access to more emotion-inducing items, shifting its underlying prediction logic from conservative to aggressive. This amplifies the model’s sensitivity overload, causing it to prioritize capturing subtle lexical features and force-fit emotional labels when processing ambiguous contexts.

(2) Arousal shift in sadness

GPT-5 can accurately discern emotional polarity (positive or negative), but it exhibits significant cognitive ambiguity when distinguishing negative emotions with identical valence but differing arousal levels. The most common bias involves misclassifying sadness as other negative emotions such as anger, fear, or disgust. The misclassifications for “sadness” across five emotion taxonomies are shown in Table 11.

**Table 11:** The misclassification for sadness across five emotion taxonomies.

Emotion taxonomy	Samples	Error rate	Main misclassification results
SemEval	436	36.01%	Sadness → fear, anger
Ekman	354	44.07%	Sadness → fear, anger, disgust
SevenEmotions	373	56.57%	Sadness → fear, anger, disgust
Plutchik	344	54.36%	Sadness → fear, anger, disgust
GoEmotions	213	87.32%	Sadness → fear, remorse, disappointment, annoyance

This bias reflects the model’s valency-first strategy. While the model correctly identifies negative polarity in post, it exhibits bias when distinguishing between psychological motivations such as inward-directed distress (sadness) versus outward-directed aggression (anger) or future threat (fear). It often displays emotional polarization, misclassifying low-arousal sadness as higher-arousal negative emotions.

The misclassification rate for sadness increases significantly with higher emotion granularity. In SemEval, the error rate is 36.01%, with misclassifications primarily directed toward fear and anger. In Ekman, SevenEmotions, and Plutchik, the error rate rises, peaking at 56.57% in SevenEmotions. In GoEmotions, the error rate reaches a maximum of 87.32%; beyond misclassifying as high-arousal fear, the model attempts to distinguish among several semantically overlapping emotions but becomes severely misaligned due to the lack of clear definitions for each label.

## 5 Discussion

This paper aims to address a critical gap in LLM emotion analysis: how emotion taxonomy, as a cognitive framework, shapes models’ labeling behavior. By comparing GPT-5’s behavior across five taxonomies—SemEval, Ekman, SevenEmotions, Plutchik, and GoEmotions—in Chinese Weibo emotional classification tasks, we find that emotion taxonomy is not neutral to LLM’s emotion analysis. Instead, it serves as a moderating variable that determines model performance, human-machine alignment, and bias patterns.

### 5.1 The granularity paradox

The most significant finding of this study is that the emotion granularity exhibits a strong negative correlation with GPT-5’s classification performance. As the number of emotion categories expanded from 4 to 27, the model’s performance declined significantly. This reflects the model’s distributional sensitivity to label overlaps within the zero-shot prompt.

As the taxonomy becomes highly granular (e.g., GoEmotions), the semantic distance between adjacent emotion labels in the high-dimensional embedding space decreases substantially. This creates boundary interference, where the model struggles to delineate fine-grained distinctions (e.g., sadness vs. remorse) solely through pre-trained knowledge.

Crucially, we must address whether this decline is driven by taxonomic complexity or by the scarcity of rare categories (e.g., the long-tail distribution in GoEmotions). Two lines of evidence from our study support taxonomic complexity as the primary driver:

(1) Even for samples with unanimous human agreement (CL 5), the accuracy still drops by 17.95 percentage points from SemEval to GoEmotions. This indicates that the failure stems from label space competition rather than the inherent ambiguity or rarity of the samples.
(2) The error pattern for “sadness” reveals that performance drops not because the “sadness” sample becomes rare, but because the introduction of overlapping synonyms acts as distractors. Therefore, the granularity paradox acts as a systemic penalty imposed by the crowded decision space.

This finding provides crucial boundary conditions for current optimistic expectations regarding LLM emotion analysis capabilities. Although Niu et al. (2024) and Liu et al. (2024) report GPT-4 achieving human-level performance in emotion recognition, our research indicates this human-level capability is conditional, primarily manifesting in coarse-grained frameworks (e.g., SemEval). These findings challenge overly optimistic expectations of LLM zero-shot omnipotence. This performance degradation is not solely driven by increased category numbers but stems from deeper causes like semantic overlap. This aligns with findings from human annotation studies (Demszky et al., 2020; Bostan and Klinger, 2018): fine-grained classification forces models to make binary splits between highly similar concepts, leading to increased entropy in the model’s internal probability distribution.

Interestingly, we observed an anomalous increase in recognition rates for anger in GoEmotions. This suggests that when a fine-grained taxonomy successfully isolates variants of an emotion (e.g., annoyance), the semantic purity of the core label can enhance the model’s focus. This offers new insights for future prompt engineering: mitigating the granularity paradox through hierarchical classification rather than flattened classification.

### 5.2 Human-AI alignment gap

This study reveals significant discrepancies between GPT-5 and human annotators (McNemar test p<0.001). Regarding group consistency, incorporating GPT-5 slightly reduced consistency. While the Granularity Paradox—where performance degrades as the decision space expands—remains robust for GPT-5, our analysis uncovers a divergent trend for human annotators. Specifically, as emotion taxonomies grow more complex, human inter-annotator agreement does not decline as predicted but remains stable.

Our investigation into this phenomenon indicates that the high human consensus level in fine-grained contexts is not a result of sample selection bias, as the NC exclusion rates remained consistent across all taxonomies. Instead, it highlights a fundamental difference in cognitive strategies:

In broad taxonomies, humans often grapple with “boundary ambiguity” (e.g., whether a subtle emotion fits into the wide “joy” category). However, fine-grained taxonomies provide semantic specificity (e.g., gratitude, remorse). These precise labels act as clearer cognitive anchors, allowing human annotators to reach consensus through intuitive cue matching grounded in life experience.

In contrast, GPT-5 increasingly struggles to replicate human consensus as granularity increases, evidenced by the decline in Fleiss’ kappa when the model is incorporated. This suggests that while humans benefit from the clarity of specific definitions, LLMs become more susceptible to the noise of probabilistic co-occurrence within expanding label space.

Consequently, the significant divergence in cognitive pathways is most pronounced in fine-grained contexts: humans leverage semantic precision to maintain collective agreement, whereas the model’s predictive stability is challenged by the high-dimensional complexity of nuanced emotions.

Regarding cultural adaptability, this study observed a counterintuitive phenomenon: despite the Seven Emotions theory originating from Chinese culture and theoretically aligning better with Chinese Weibo, GPT-5’s performance under this taxonomy was not superior to—and even slightly inferior to—the Ekman taxonomy. This finding challenges the simplistic assumption that “native labels equate to native understanding,” revealing cultural schema bias within LLMs. Although GPT-5 ingested Chinese data during pre-training and can grasp the literal meanings of terms like love (爱) and desire (欲), its underlying emotional cognitive schema remains dominated by mainstream Western psychological frameworks (e.g., the Ekman/Plutchik model). When processing uniquely Chinese emotional concepts, the model may need to internally map them to proximate Western concepts (e.g., mapping “欲” to “desire”). Semantic loss during this mapping process negates the potential advantages of localized labels. This aligns with Nasution et al. (2025) that found LLMs struggle in non-English contexts, suggesting that simple label replacement is insufficient to bridge cultural divides. Future localization research must focus on deep semantic alignment.

### 5.3 Classification bias

This study identifies robust classification biases in GPT through confusion matrices, revealing underlying flaws in LLM emotional recognition. It validates and extends (Troiano et al., 2023) on annotation tool-induced bias.

First, over-interpretation of “neutral” post. Findings indicate that as emotion taxonomy grows more complex, models increasingly misclassify neutral text as emotional (rising from 28.4% in SemEval to 45.4% in GoEmotions). This “emotional hallucination” likely stems from LLMs’ instruction-following bias: when prompts provide extensive emotion lists, models tend to over-capture weak lexical cues in text and force emotional labels onto them.

Second, the “arousal shift” of low-arousal negative emotions. Models frequently misclassify sadness as fear or anger. Anger and fear typically exhibit more pronounced biological markers and linguistic intensity (e.g., exclamation marks, aggressive vocabulary). GPT-5 appears to have learned a heuristic strategy prioritizing valence over intensity: after determining text is negative, it tends to classify it as a higher-arousal emotion that is more common and more distinctively featured in the training data. This bias is amplified in complex taxonomies, as fine-grained labels dilute the defining boundaries of original categories, causing the model to regress toward stronger prototypical emotions.

Based on these findings, the following recommendations are proposed for affective computing using LLMs:

- Occam’s Razor Principle: Prioritize coarse-grained taxonomies like SemEval or Ekman. A simple ruler yields more robust performance and higher human-machine alignment.
- Calibrating Neutral Thresholds: Given LLMs’ tendency to emotionalize neutral text, deployments should implement dedicated neutral filters or explicitly weight emotionless judgments in prompts.
- Cultural Awareness Enhancement: For non-English contexts, directly applying localized taxonomies (e.g., SevenEmotions) may not directly improve LLM performance. Future research should explore injecting specific cultural cognitive schemas into models through few-shot learning or Chain-of-Thought (CoT) techniques to mitigate pretraining biases.

## 6 Conclusion

This study empirically compared GPT’s emotion annotation behavior across five emotion taxonomies (SemEval, Ekman, SevenEmotions, Plutchik, and GoEmotions) to deeply explore the impact of emotion taxonomy on GPT-5. Findings confirm that emotion taxonomies are not neutral task contexts but core variables that modulate LLM annotation performance, human-machine consistency, and bias characteristics.

First, a significant negative correlation exists between emotion granularity and model performance. As taxonomy transition from coarse to fine granularity, GPT-5’s recognition efficacy demonstrably declines. This “granularity paradox” indicates that LLM affective computing heavily relies on task space definition, where semantic space overcrowding significantly increases the model’s discriminative load. Furthermore, the localized SevenEmotions failed to demonstrate expected cultural adaptability advantages, revealing underlying Western-centric biases in the model’s deep cognitive schemas.

Second, emotion taxonomy is a key factor influencing human-machine alignment. Experiments show statistically significant differences between GPT-5 and human annotators across all emotion taxonomies, with this cognitive dissonance intensifying as taxonomy complexity increases. This reveals deep-seated deviations between the model’s judgment of complex semantic boundaries and human logic.

Third, this study identifies two robust cross-taxonomy biases: first, a tendency toward emotional overinterpretation, where models erroneously emotionalize neutral text by capturing extremely subtle lexical cues; second, the negative emotion collapse phenomenon, manifested as low-arousal emotions like sadness logically shifting toward high-arousal prototypes such as fear and anger.

## 7 Limitation

This study delineates the boundaries of LLM emotional cognition across diverse emotion taxonomies, providing empirical support for constructing robust automated emotion annotation paradigms. However, several limitations must be acknowledged.

First, our evaluation is primarily based on GPT-5. It remains to be determined whether the observed performance degradation in fine-grained taxonomies is universally shared by LLMs. Future research should extend this evaluation across a broader spectrum of open-source and proprietary LLMs to validate these findings.

Second, this study focuses on zero-shot capabilities of GPT-5 without employing few-shot learning, Chain-of-Thought (CoT) reasoning, or advanced prompt optimization. As taxonomic complexity increases, these techniques might help mitigate semantic entropy and systemic biases, though exploring such interventions was beyond our current scope.

Third, our findings are based on Sina Weibo posts. The linguistic idiosyncrasies of Weibo—such as informal syntax and culture-specific internet slang—differ significantly from formal texts or Western social media (e.g., Twitter/X). Consequently, the observed effects may not be directly transferable to other linguistic contexts or cross-cultural emotion recognition tasks.

Fourth, although the dataset contains 2,848 posts, the sample distribution becomes increasingly fragmented as taxonomic granularity increases. In fine-grained taxonomies, the data exhibits a pronounced long-tail distribution, where certain categories contain limited samples. Future studies should aim for more balanced datasets to disentangle the effects of taxonomic complexity from data scarcity.

Fifth, this study adopted a single-query approach for each post. As noted in Komatsu et al. (2022), single-pass annotation may fail to capture the model’s inherent stochastic variability. Future research should employ multi-sampling techniques to provide a more robust assessment of LLM in affective tasks.

## Data Availability

The original contributions presented in the study are included in the article/Supplementary material, further inquiries can be directed to the corresponding author.

## References

[ref1] Altun S. N. Dörterler M. (2025). A systematic testbed for evaluating emotion classification in large language models. J. Sci. Rep. B 13, 1–19. Available online at: https://izlik.org/JA98MA47ZB

[ref2] Amin M. M. Mao R. Cambria E. Schuller B. W. (2024). A wide evaluation of ChatGPT on affective computing tasks. IEEE Trans. Affect. Comput. 15, 2204–2212. doi: 10.1109/TAFFC.2024.3419593

[ref3] Aragón M. E. López-Monroy A. P. González-Gurrola L. C. Montes-y-Gómez M. (2023). Detecting mental disorders in social media through emotional patterns—the case of anorexia and depression. IEEE Trans. Affect. Comput. 14, 211–222. doi: 10.1109/TAFFC.2021.3075638

[ref4] Baddeley A. (2003). Working memory: looking back and looking forward. Nat. Rev. Neurosci. 4, 829–839. doi: 10.1038/nrn1201, 14523382

[ref5] Bagdon C. Karmalker P. Gurulingappa H. Klinger R. (2024). “You are an expert annotator”: automatic best-worst-scaling annotations for emotion intensity modeling. NAACL 1, 7924–7936. doi: 10.48550/ARXIV.2403.17612

[ref6] Belay T. D. Azime I. A. Ayele A. A. Sidorov G. Klakow D. Slusallek P. . (2025). Evaluating the capabilities of large language models for multi-label emotion understanding. In Rambow O. Wanner L. Apidianaki M. Al-Khalifa H. Eugenio B. D. Schockaert S. (Eds.), Proceedings of the 31st International Conference on Computational Linguistics (pp. 3523–3540). Abu Dhabi, UAE: Association for Computational Linguistics.

[ref7] Bojic L. Zagovora O. Zelenkauskaite A. Vukovic V. Cabarkapa M. Jerkovic S. V. . (2025). Evaluating large language models against human annotators in latent content analysis: sentiment, political leaning, emotional intensity, and sarcasm. arXiv. doi: 10.48550/arXiv.2501.02532 PMC11968858 40181141

[ref8] Bostan L.-A.-M. Klinger R. (2018). An analysis of annotated corpora for emotion classification in text. Proceedings of the 27th International Conference on Computational Linguistics, 2104–2119.

[ref9] Brady M. (2021). Précis: emotions: the basics. J. Philo. Emotion 3, 1–4. doi: 10.33497/2021.summer.1

[ref10] Chakraborty K. Bhattacharyya S. Bag R. (2020). A survey of sentiment analysis from social media data. IEEE Trans. Comput. Soc. Syst. 7, 450–464. doi: 10.1109/TCSS.2019.2956957

[ref11] Chu M. Song W. Zhao Z. Chen T. Chiang Y. (2024). Emotional contagion on social media and the simulation of intervention strategies after a disaster event: a modeling study. Humanit. Soc. Sci. Commun. 11:968. doi: 10.1057/s41599-024-03397-4

[ref12] De León Languré A. Zareei M. (2024). Evaluating the effect of emotion models on the generalizability of text emotion detection systems. IEEE Access 12, 70489–70500. doi: 10.1109/ACCESS.2024.3401203

[ref13] Demszky D. Movshovitz-Attias D. Ko J. Cowen A. Nemade G. Ravi S. (2020). GoEmotions: a dataset of fine-grained emotions. Proceedings of the 58th Annual Meeting of the Association for Computational Linguistics, Eds. D. Jurafsky, J. Chai, N. Schluter, J. Tetreault, 4040–4054

[ref14] Ekman P. (1992). An argument for basic emotions. Cogn. Emot. 6, 169–200. doi: 10.1080/02699939208411068

[ref15] Field A. Park C. Y. Theophilo A. Watson-Daniels J. Tsvetkov Y. (2022). An analysis of emotions and the prominence of positivity in #BlackLivesMatter tweets. Proc. Natl. Acad. Sci. 119:e2205767119. doi: 10.1073/pnas.2205767119, 35998217 PMC9436370

[ref16] Greschner L. Klinger R. (2025). Fearful falcons and angry llamas: emotion category annotations of arguments by humans and LLMs. arXiv. doi: 10.48550/arXiv.2412.15993

[ref17] Harmon-Jones E. Harmon-Jones C. Summerell E. (2017). On the importance of both dimensional and discrete models of emotion. Behav. Sci. 7:66. doi: 10.3390/bs7040066, 28961185 PMC5746675

[ref18] Huang J. Jiao W. Lam M. Li E. Lyu M. Ren S. . (2024). “Apathetic or empathetic? Evaluating LLMs’ emotional alignments with humans,” in Advances in Neural Information Processing Systems. Eds. D. Belgrave, A. Fan, U. Paquet, J. Tomczak, C. Zhang, vol. 37 (97053–97087).

[ref19] Kaddour J. Harris J. Mozes M. Bradley H. Raileanu R. McHardy R. (2023). Challenges and applications of large language models. arXiv. doi: 10.48550/arXiv.2307.10169

[ref20] Ke H. Watanabe E. (2025). Does GPT-4 decode richer architecture of emotion? Mapping dimensional structure with 99 emotions. Open Science Framework. doi: 10.31219/osf.io/sdnbh_v2

[ref21] Komatsu H. Maeno A. Watanabe E. (2022). Origin of the ease of association of color names: comparison between humans and AI. I-Perception 13:20416695221131832. doi: 10.1177/20416695221131832, 36330043 PMC9623380

[ref22] Koptyra B. Ngo A. Radliński Ł. Kocoń J. (2023). “CLARIN-emo: training emotion recognition models using human annotation and ChatGPT,” in Lecture Notes in Computer Science, Eds. J. Mikyška, C. de Mulatier, M. Paszynski, V. V. Krzhizhanovskaya, J. J. Dongarra, P. M. Sloot (Brague, Czech Republic: Springer Nature Switzerland), 365–379.

[ref23] Kumar S. Prabha R. Samuel S. (2022). “Sentiment analysis and emotion detection with healthcare perspective,” in Augmented Intelligence in Healthcare: A pragmatic and Integrated Analysis, eds. Mishra S. Tripathy H. K. Mallick P. Shaalan K., vol. 1024 (Singapore: Springer Nature Singapore), 189–204.

[ref24] Landis J. R. Koch G. G. (1977). The measurement of observer agreement for categorical data. Biometrics 33:159. doi: 10.2307/2529310, 843571

[ref25] Lerner J. S. Li Y. Valdesolo P. Kassam K. S. (2015). Emotion and decision making. Annu. Rev. Psychol. 66, 799–823. doi: 10.1146/annurev-psych-010213-115043, 25251484

[ref26] Li Y. Peng L. Sang Y. Gao H. (2024). The characteristics and functionalities of citizen-led disaster response through social media: a case study of the #HenanFloodsRelief on Sina Weibo. Int. J. Disaster Risk Reduct. 106:104419. doi: 10.1016/j.ijdrr.2024.104419

[ref27] Liu Z. Yang K. Xie Q. Zhang T. Ananiadou S. (2024). EmoLLMs: a series of emotional large language models and annotation tools for comprehensive affective analysis. Proceedings of the 30th ACM SIGKDD Conference on Knowledge Discovery and Data Mining, 5487–5496

[ref28] Mohammad S. Bravo-Marquez F. Salameh M. Kiritchenko S. (2018). SemEval-2018 task 1: affect in tweets. Proceedings of the 12th International Workshop on Semantic Evaluation, 1–17

[ref29] Nasution A. H. Onan A. Murakami Y. Monika W. Hanafiah A. (2025). Benchmarking open-source large language models for sentiment and emotion classification in Indonesian tweets. IEEE Access 13, 94009–94025. doi: 10.1109/ACCESS.2025.3574629

[ref30] Niu M. Jaiswal M. Mower Provost E. (2024). From text to emotion: unveiling the emotion annotation capabilities of LLMs. Interspeech, 2650–2654. doi: 10.21437/interspeech.2024-2282

[ref31] Ortony A. Clore G. L. Collins A. (1990). The Cognitive Structure of Emotions. Cambridge, UK: Cambridge University Press.

[ref32] Parrott W. G. (2001). Emotions in Social Psychology: Key Readings. London, UK: Psychology Press. Available online at: https://app.readcube.com/library/f7ea0e70-5953-4fb0-b352-463ff128bed6/item/9d01351b-ea71-4385-9ea7-bf58b92073ba

[ref33] Plutchik R. (2001). The nature of emotions: human emotions have deep evolutionary roots, a fact that may explain their complexity and provide tools for clinical practice. Am. Sci. 89:344. doi: 10.1511/2001.4.344

[ref34] Qi H. Zhao Q. Li J. Song C. Zhai W. Luo D. . (2023). Supervised learning and large language model benchmarks on mental health datasets: cognitive distortions and suicidal risks in Chinese social media. arXiv. doi: 10.48550/ARXIV.2309.03564 PMC12383806 40868395

[ref35] Scherer K. R. Wallbott H. G. (1994). Evidence for universality and cultural variation of differential emotion response patterning. J. Pers. Soc. Psychol. 66, 310–328. doi: 10.1037/0022-3514.66.2.310, 8195988

[ref36] Sheng Dai. (2013). The Book of Rites (Li ji). Createspace Independent Pub. Scotts Valley, California, US. Available online at: https://app.readcube.com/library/f7ea0e70-5953-4fb0-b352-463ff128bed6/item/3499dd15-761f-4b2a-b7af-55ff0c395eba

[ref37] Singh A. Fry A. Perelman A. Tart A. Ganesh A. El-Kishky A. . (2025). OpenAI GPT-5 system card. arXiv. doi: 10.48550/arXiv.2601.03267

[ref38] Sweller J. (1988). Cognitive load during problem solving: effects on learning. Cogn. Sci. 12, 257–285. doi: 10.1207/s15516709cog1202_4

[ref39] Thapa S. Shiwakoti S. Shah S. B. Adhikari S. Veeramani H. Nasim M. . (2025). Large language models (LLM) in computational social science: prospects, current state, and challenges. Soc. Netw. Anal. Min. 15:4. doi: 10.1007/s13278-025-01428-9

[ref40] Tohidi K. Dashtipour K. Rebora S. Pourfaramarz S. (2025). A comparative evaluation of large language models for Persian sentiment analysis and emotion detection in social media texts. arXiv. doi: 10.48550/arXiv.2509.14922

[ref41] Troiano E. Oberlander L. Klinger R. (2023). Dimensional modeling of emotions in text with appraisal theories: corpus creation, annotation reliability, and prediction. Comput. Ling. 49, 1–72 (WOS:000993797000001). doi: 10.1162/coli_a_00461

[ref42] Wang Y. (2020). The Three-character classic. People’s Literature Publishing House. Available online at: https://app.readcube.com/library/f7ea0e70-5953-4fb0-b352-463ff128bed6/item/2dfe24e5-5afb-47a3-9936-7614477228fe

[ref43] Williams L. Arribas-Ayllon M. Artemiou A. Spasic I. (2019). Comparing the utility of different classification schemes for emotive language analysis. J. Classif. 36, 619–648. doi: 10.1007/s00357-019-9307-0

[ref44] Zhang F. Chen J. Tang Q. Tian Y. (2024). Evaluation of emotion classification schemes in social media text: an annotation-based approach. BMC Psychol. 12:503. doi: 10.1186/s40359-024-02008-w, 39334344 PMC11438282

[ref45] Zhang W. Deng Y. Liu B. Pan S. Bing L. (2024). “Sentiment analysis in the era of large language models: a reality check,” in Findings of the Association for Computational Linguistics: NAACL 2024, eds. Duh K. Gomez H. Bethard S. (Mexico City, Mexico: Association for Computational Linguistics), 3881–3906.

[ref46] Zhang F. Tang Q. Chen J. Han N. (2023). China public emotion analysis under normalization of COVID-19 epidemic: using Sina Weibo. Front. Psychol. 13:1066628. doi: 10.3389/fpsyg.2022.1066628, 36698592 PMC9870544

