# Supplementary material for: Granularity paradox: how emotion taxonomies shape GPT-5’s affective cognition and human-AI alignment
Source: Front Psychol. 2026 Mar 13;17:1786724. doi: 10.3389/fpsyg.2026.1786724 (PMC13021436; doi:10.3389/fpsyg.2026.1786724)
Supplement: Supplementary file 1 [file Table_1.docx]

# Appendix

Table S1. Label distribution for the SemEval taxonomy

| Emotion Label | Sample Count | Percentage |
| --- | --- | --- |
| joy | 252 | 11.0% |
| anger | 151 | 6.6% |
| sadness | 438 | 19.2% |
| fear | 101 | 4.4% |
| neutral | 1339 | 58.7% |
| Total | 2281 | 100% |

Table S2. Label distribution for the Ekman taxonomy

| Emotion Label | Sample Count | Percentage |
| --- | --- | --- |
| joy | 193 | 8.3% |
| anger | 118 | 5.1% |
| sadness | 483 | 20.7% |
| fear | 119 | 5.1% |
| surprise | 57 | 2.4% |
| disgust | 8 | 0.3% |
| neutral | 1353 | 58.0% |
| Total | 2331 | 100% |

Table S3. Label distribution for the SevenEmotions taxonomy

| Emotion Label | Sample Count | Percentage |
| --- | --- | --- |
| joy | 200 | 8.4% |
| anger | 108 | 4.5% |
| sadness | 464 | 19.5% |
| fear | 132 | 5.5% |
| love | 17 | 0.7% |
| disgust | 24 | 1.0% |
| desire | 11 | 0.5% |
| neutral | 1425 | 59.8% |
| Total | 2381 | 100% |

Table S4. Label distribution for the Plutchik taxonomy

| Emotion Label | Sample Count | Percentage |
| --- | --- | --- |
| joy | 187 | 7.6% |
| anger | 101 | 4.1% |
| sadness | 470 | 19.2% |
| fear | 118 | 4.8% |
| disgust | 29 | 1.2% |
| surprise | 72 | 2.9% |
| trust | 3 | 0.1% |
| anticipate | 22 | 0.9% |
| neutral | 1450 | 59.1% |
| Total | 2452 | 100% |

Table S5. Label distribution for the GoEmotions taxonomy

| Emotion Label | Sample Count | Percentage |
| --- | --- | --- |
| Admiration | 14 | 0.6% |
| Amusement | 40 | 1.6% |
| Anger | 111 | 4.4% |
| Annoyance | 26 | 1.0% |
| Approval | 45 | 1.8% |
| Caring | 29 | 1.1% |
| Confusion | 106 | 4.2% |
| Curiosity | 39 | 1.5% |
| Desire | 29 | 1.1% |
| Disappointment | 14 | 0.6% |
| Disapproval | 9 | 0.4% |
| Disgust | 2 | 0.1% |
| Embarrassment | 7 | 0.3% |
| Excitement | 29 | 1.1% |
| Fear | 95 | 3.7% |
| Gratitude | 18 | 0.7% |
| Grief | 7 | 0.3% |
| Joy | 83 | 3.3% |
| Love | 17 | 0.7% |
| Nervousness | 51 | 2.0% |
| Optimism | 71 | 2.8% |
| Pride | 8 | 0.3% |
| Realization | 9 | 0.4% |
| Relief | 3 | 0.1% |
| Remorse | 4 | 0.2% |
| Sadness | 396 | 15.6% |
| Surprise | 22 | 0.9% |
| Neutral | 1252 | 49.4% |
| Total | 2536 | 100% |

Table S6. Within-annotator self-consistency

| Annotators | Ratio of Consistency | Cases | Count of Consistency |
| --- | --- | --- | --- |
| Annotator 1 | 67.61% | 2692 | 1820 |
| Annotator 2 | 96.38% | 2710 | 2612 |
| Annotator 3 | 83.79% | 2141 | 1794 |
| Annotator 4 | 59.61% | 2748 | 1638 |
| Annotator 5 | 93.23% | 2142 | 1997 |

Table S7. Average pairwise Cohen’s kappa for individual annotators across five taxonomies

| Annotators | SemEval | Ekman | SevenEmotions | Plutchik | GoEmotions |
| --- | --- | --- | --- | --- | --- |
| Annotator 1 | 0.423 | 0.367 | 0.383 | 0.392 | 0.516 |
| Annotator 2 | 0.459 | 0.376 | 0.367 | 0.389 | 0.505 |
| Annotator 3 | 0.358 | 0.339 | 0.346 | 0.342 | 0.265 |
| Annotator 4 | 0.403 | 0.238 | 0.308 | 0.316 | 0.281 |
| Annotator 5 | 0.412 | 0.366 | 0.361 | 0.389 | 0.430 |
